# Hypothermia Mitigates Renal Fibrosis Through the Upregulation of PGC-1α After Ischemia–Reperfusion Injury

**DOI:** 10.3390/biomedicines13061337

**Published:** 2025-05-29

**Authors:** Dabi Kim, Suyeon Han, Hyunsu Choi, Yoon-Kyung Chang, Dae Eun Choi

**Affiliations:** 1 Department of Medical Science, Medical School, Chungnam National University, Daejeon 35015, Republic of Korea; dabi941012@gmail.com; 2 Department of Nephrology, Daejeon Saint Mary’s Hospital, Catholic University of Korea, Daejeon 34943, Republic of Korea; sooyaa1208@gmail.com; 3 Clinical Research Institute, Daejeon Saint Mary’s Hospital, Catholic University of Korea, Daejeon 34943, Republic of Korea; peace420@cmcdj.or.kr; 4 Department of Nephrology, Medical School, Chungnam National University, Daejeon 35015, Republic of Korea

**Keywords:** hypothermic protection, renal fibrosis, ischemia–reperfusion injury

## Abstract

**Background:** Hypothermia has been previously reported to ameliorate acute renal injury induced by ischemia–reperfusion injury (IRI). However, its protective effects against subsequent renal fibrosis remain unclear. **Objectives**: The aim of this study was to determine whether hypothermia provides protection against renal ischemia–reperfusion injury (IRI), and to elucidate the molecular mechanisms involved. **Methods**: We used a model of renal fibrosis after ischemia–reperfusion injury in mice. C57BL/6 mice were divided into the following groups: control mice and ischemia–reperfusion injury mice (at 37 °C and at 32 °C). Their kidneys were harvested on day 1, day 3, and day 7 after IRI. The molecular mechanisms were evaluated. **Results**: The blood urea nitrogen (BUN) levels, serum creatinine (s-Cr) levels, and the histologic renal injury scores were significantly lower in the 32 °C IRI group than in the 37 °C ischemia–reperfusion injury group. In the hypothermic IR group, TGF-β and α-SMA were significantly decreased, while the PGC-1α level was significantly increased. Cold preparation increased the PGC-1α levels in HK2 cells. In TGF-β-treated HK2 cells, cold preparation decreased α-SMA and collagen IV levels. In addition, siPGC-1α in HK2 cells increased α-SMA and collagen IV, despite cold preparation. **Conclusions**: Hypothermia attenuates renal function deterioration and renal fibrosis in renal IRI mice kidneys. PGC-1α may play a role in hypothermic protection in renal fibrosis after IRI.

## 1. Introduction

Chronic kidney disease (CKD) is a major clinical issue that requires renal replacement therapy, dialysis, and kidney transplantation. It is noteworthy that there is increasing evidence indicating an association of renal ischemia–reperfusion injury (IRI) with renal fibrosis that progresses to end-stage renal disease (ESRD) [1,2]. Also, renal IRI is considered to be a pathological mechanism of renal injury that is inevitable in organ transplantations [1,3,4]. Renal ischemic injury results in oxygen deficiency, mitochondria permeability changes, and cellular apoptosis. In addition, reperfusion injury leads to the extensive generation of reactive oxygen species (ROS), inflammatory cytokine, and chemokines. Thus, renal IRI causes tubular cell necrosis and apoptosis and tubulointerstitial inflammation [5]. After renal IRI, regeneration and recovery of the renal tubular cell occurs. However, if the renal repair process is maladaptive, it progresses to renal fibrosis. Fibrosis is induced by the crosstalk of many cell types, including epithelial, endothelial, and inflammatory cells that cause and maintain fibrosis, as well as mesenchymal cells, such as fibroblasts, pericytes, and myofibroblasts that execute fibrosis [6,7].

Hypothermic protection, the maintenance of organ temperature at 2–8 °C lower than the body cavity temperature, or experimental cooling at around 32 °C is highly protective against cellular damage and organ failure including brain injury, ischemic stroke, myocardial infarction, cardiac arrest, and kidney transplantation [8,9,10,11,12]. Our previous report showed that hypothermic protection attenuates cellular apoptosis and necrosis in acute insults. However, its effect on fibrosis or the existence of chronic change after acute results is unclear.

Generally, hypothermia is known to affect molecular reaction rates, active ion transport and ion homeostasis, energy metabolism, membrane fluidity and function, and the secretion of hormones and neurotoxins [13]. Although several proteins, including cold-inducible RNA-binding protein (CIRBP), RNA-binding motif protein 3 (RBM3), AMPK, ERK, HIF1, and peroxisome proliferator-activated receptor-γ coactivator (PGC)-1α, are induced and play a role in protection from organ injury [14,15,16,17,18], much remains unclear regarding which molecular mechanisms are involved in hypothermic protection [10]. Although recent studies have also demonstrated that hypothermia can attenuate fibrogenic pathways including TGF-β/smad signaling in the brain [19], little is known about the role of hypothermia in various chronic organ injuries or in fibrosis.

The aim of this study was to evaluate whether hypothermia could improve renal fibrosis after renal IRI in mice and to study which molecular mechanisms are involved in hypothermic protection of the kidneys.

## 2. Materials and Methods

### 2.1. Animal Model

**Figure 1:**
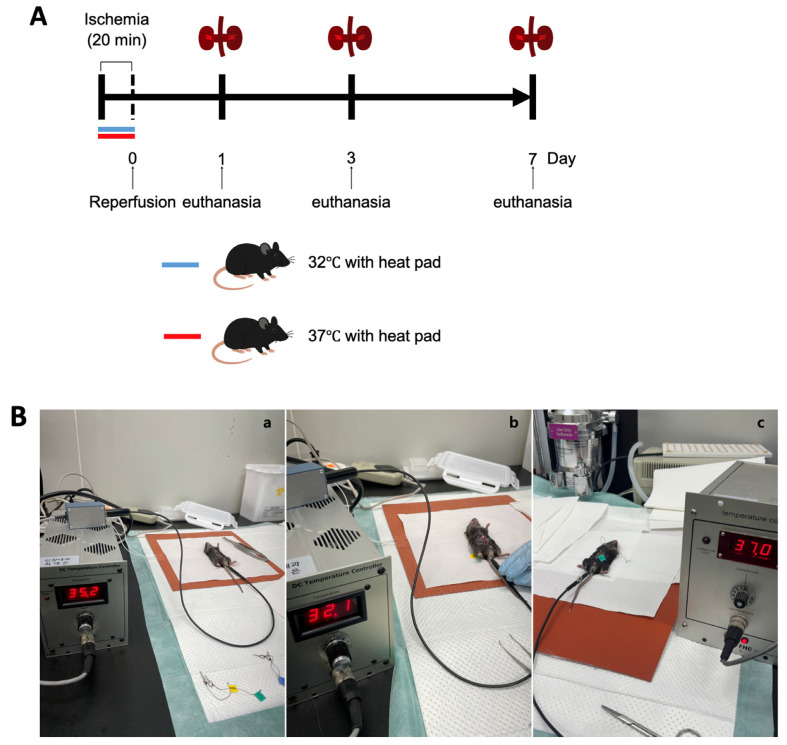
Experimental design and procedure for ischemia–reperfusion (IR) model under hypothermic and normothermic conditions. (**A**) Mice were randomly assigned to either a normothermia group (core temperature maintained at 37 °C using a heating pad) or a hypothermia group (core temperature maintained at 32 °C using a heating pad) during the IR procedure. All animals underwent 20 min of bilateral renal ischemia, followed by reperfusion. Mice were sacrificed at 1, 3, or 7 days post-reperfusion for sample collection. (**B**) Experimental setup for core temperature regulation and IR surgery. (**a**) Mice were anesthetized and placed on a heating pad system equipped with a digital temperature controller in an SPF room maintained at 26 ± 1 °C. Abdominal incisions were made to expose the renal pedicles. (**b**) In the hypothermic group, core temperature was stably maintained at 32 ± 1 °C within 5 min of positioning the animal on the pad. Once the desired temperature was reached, ischemia was induced by clamping both renal pedicles with microvascular clips for 20 min. The clips were then removed to allow for reperfusion. (**c**) In the normothermic group, body temperature was stabilized at 37 ± 1 °C under identical surgical and temperature control conditions.

Male C57BL/6 mice aged 10 weeks were obtained from Samtako Bio Korea, located in Gyeonggi-do, South Korea. The animal care and handling followed guidelines approved by the Institutional Animal Care and Use Committee at Chungnam National University Medical School (approval number: CNU-01180). Mice were categorized based on environmental temperature conditions at 32 °C and 37 °C, and subsequently divided into groups evaluated on days 1, 3, and 7 post-ischemia–reperfusion injury (IRI) (Figure 1A).

The procedure to induce renal IRI was conducted as previously detailed. In short, mice received anesthesia through an intraperitoneal injection containing a mixture of ketamine (60 mg/kg body weight) and xylazine (8 mg/kg body weight). A surgical incision was made in the abdomen, and bilateral renal pedicles were gently clamped to restrict blood flow. During surgery, the body temperatures of the mice were maintained precisely at either 32 °C or 37 °C, respectively. To maintain the target consistent core temperature during surgery, mice were housed in a specific pathogen-free experimental facility (Preclinical Rodent Facility of Chungam National University Hospital, room temperature 23 ± 1 °C). Under anesthesia, abdominal incisions were made to expose the renal pedicles. The animals were assigned to either the hypothermic or normothermic group. Mice were placed on a heating pad system equipped with a digital temperature controller and connected to a heating pad (FHC, Bowdoin, ME, USA; Cat# FHC 40-90-2). Rectal temperature was continuously monitored using a Mini Rectal Thermistor Probe (FHC, Bowdoin, ME, USA; Cat# FHC 40-90-5D-02) and automatically regulated with a DC Temperature Controller (FHC, Bowdoin, ME, USA; Cat# FHC 40-90-8D). This setup successfully maintained the mice’s rectal temperatures at 37 ± 1 °C and 32 ± 1 °C, respectively. In the hypothermic group, the core temperature was maintained at 32 ± 1 °C within five minutes of positioning the animal on the pad. Once the target temperature was reached, ischemia was induced by clamping both renal pedicles with microvascular clips for 20 min, after which the clips were removed to allow for reperfusion. In the normothermic group, body temperature was stabilized at 37 ± 1 °C under the same surgical and temperature control conditions. The ischemia and reperfusion procedure was conducted identically to that in the hypothermic group (Figure 1B).

Mice were euthanized at designated intervals (on days 1, 3, and 7 after surgery), and samples of blood and kidney tissues were harvested for subsequent analysis (Figure 1A). Throughout the study duration, none of the animals exhibited mortality, discomfort, or distress. Euthanasia involved administering ketamine anesthesia, followed by cervical dislocation for humane termination.

Hypothermia protection (32 °C) was performed during ischemia. Reperfusion was performed 20 min after ischemia. The mice’s kidneys and blood were collected 1 day, 3 days, and 7 days after reperfusion for the evaluation of renal fibrosis and renal function.

### 2.2. Blood and Tissue Preparation

Blood was drawn from the inferior vena cava immediately following euthanasia and transferred to pre-cooled Eppendorf tubes maintained at 4 °C. Serum separation was performed by centrifuging samples at 4 °C for 20 min, after which aliquots of serum were rapidly frozen in liquid nitrogen and preserved at −70 °C for later use. Kidney tissues were processed as previously detailed. Briefly, kidneys were removed promptly post-mortem and divided into three coronal segments. Two of these segments were snap-frozen with liquid nitrogen and stored at −70 °C for RNA and protein studies. The remaining section of each kidney was fixed in 4% paraformaldehyde at 4 °C and embedded in paraffin blocks for subsequent immunohistochemical analyses.

### 2.3. Light Microscopy

Paraffin-embedded kidney tissues were sectioned at a thickness of 4 μm and placed onto glass microscope slides. Following deparaffinization with xylene, sections underwent staining with hematoxylin–eosin (H and E) and Periodic Acid–Schiff (PAS) dyes, after which they were analyzed microscopically using an Olympus BX51 microscope (Olympus, Tokyo, Japan). In the H and E-stained sections, pathological features including cortical vacuolization, the infiltration of leukocytes in the peritubular and proximal tubular regions, and the simplification of the proximal tubules were assessed and scored using the following criteria: 0 represented normal morphology, 1 indicated mild injury, 2 corresponded to moderate injury, and 3 denoted severe injury.

Renal fibrosis was quantified via morphometric analysis employing an optical microscope coupled with a digital image analyzer system (Image-Pro plus 5.3, Mediacybernetics, Bethesda, MD, USA). Areas positively stained blue, indicative of fibrosis, were measured and quantified using computerized morphometric techniques. The evaluation of kidney sections was performed by a trained pathologist without knowledge of sample groupings to eliminate bias. For each slide, at least five microscopic fields were observed and analyzed at ×200 magnification (*n* = 5 per group).

### 2.4. Cell Culture and TGF-β1 Treatment

HK2 (human kidney proximal tubular cell-2) cells were maintained in DMEM/F-12 medium (Lonza, Walkersville, MD, USA) containing L-Glutamine and 15 mM Hepes, with 10% FBS and 1% Anti-Anti at 37 °C in 5% CO_2_. The HK-2 cells were grown; thereafter, the HK-2 cells were serum-starved for 24 h at 37 °C before human recombinant TGF-β1 treatment with 10 ng/mL during 24 h under hypothermia (33 °C).

### 2.5. PGC-1α siRNA Transfection and TGF-β1 Treatment

For PGC-1α siRNA transfection, HK-2 cells maintained at 37 °C, PGC-1α siRNA (Dharmacon, Lafayette, CO, USA; Cat# L-005111-00-0005), and control siRNA (Dharmacon, Lafayette, CO, USA; Cat# D-001810-10-05) were treated at a final concentration of 20 nM for 48 h at 37 °C in 5% CO_2_, and TGF-β1 (10 ng/mL) was added over 6 h. Eventually, the HK-2 cells were harvested.

### 2.6. Real-Time RT PCR

The total RNA of the HK-2 cells was collected using TRIzol^TM^ Reagent (Thermo Fisher Scientific, Waltham, MA, USA; Cat# 15596018) as per the manufacturer’s instructions. RNA concentration and purity were assessed using Nanodrop at 260 nm and 280 nm. The following primers were used: PGC-1α, forward 5′-TGAGTCTGTATGGAGTGACATCG-3′, reverse 5′-ACTTGAGTCCACCCAGAA AGC-3′, β-actin, forward 5′-TGTTACCAACTGGGACGACA-3′, and reverse 5′-GGG GTGTTGAAGGTCTCAAA-3′. The cDNA synthesis was prepared from the total RNA using a LunaScript^TM^ RT supermix kit (NEW ENGLAND BioLabs, Ipswich, MA, USA; Cat# E3010L), and the reaction was performed by carrying out the following incubation steps: primer annealing at 25 °C for 2 min, cDNA synthesis at 55 °C for 10 min, and heat inactivation at 95 °C for 1 min. Finally, real-time PCR was carried out using SYBR Green dye (PhileKorea, Seoul, Republic of Korea; 2X Quanti Speed SYBR mix). The PCR protocol began with an initial DNA polymerase activation at 95 °C for 2 min, followed by cycles consisting of denaturation at 95 °C for 5 s, primer annealing for 20 s, and elongation at 72 °C for 60 s, with a total of 45 cycles. A Rotor-Gene 6000 thermal cycler (Corbett Research, Mortlake, Australia) was used for amplification and real-time fluorescence detection. SYBR Green fluorescence signals were quantified after each PCR cycle, and relative gene expression was calculated via the comparative Ct method (2^−ΔΔCt^), normalizing the target gene’s Ct values against β-actin, with sham-treated mice serving as baseline controls.

### 2.7. Immunohistochemistry

Kidney tissue sections (4 μm thick) were prepared using a microtome, placed on glass slides, and heated in an oven at 60 °C for approximately one hour. Paraffin removal was performed by immersing slides in xylene for 30 min, followed by gradual rehydration through a graded ethanol series (100%, 90%, 80%, and 70%; 2 min per step), and concluding with a 10 min rinse in distilled water.

To enhance antigen detection, heat-mediated antigen retrieval was conducted by immersing slides in citrate buffer solution (Antigen Retrieval Solution, pH 6.0, Sigma Aldrich, St. Louis, MO, USA; Cat# C9999) and microwaving them for 30 min, followed by a 15 min cooldown at an ambient temperature and washing with phosphate-buffered saline (PBS). Endogenous peroxidase activity was neutralized using a 3% hydrogen peroxide solution for 15 min, after which slides underwent two washes in PBS. To prevent nonspecific antibody binding, slides were incubated with serum-free blocking solution (Dako, Glostrup, Denmark; Cat# X0909) for 20 min.

Sections were incubated overnight at room temperature with primary antibodies against TGF-β1 (Abcam, Cambridge, UK; Cat# ab64715), α-SMA (Abcam, Cambridge, UK; Cat# ab5694), collagen IV (Abcam, Cambridge, UK; Cat# ab6586), and fibronectin (Abcam, Cambridge, UK; Cat# ab2413), diluted with background reducing components (Dako, Glostrup, Denmark; Cat# S3022) overnight at room temperature; therefore, the secondary antibody was diluted with Envision + System-HRP Labeled Polymer (Dako, Glostrup, Denmark; Cat# K4003) after washing with PBS buffer. The slides were then incubated with Liquid DAB + Substrate Chromogen System (Dako, Glostrup, Denmark; Cat# K3468) for 1–10 min. After washing in distilled water, the slides were stained with Mayer’s Hematoxylin for 30 s, and washed in tap water for 10 min. Finally, the slides were dehydrated in absolute ethyl alcohol (four incubations of 2 min) followed by xylene for 30 min. The slides were mounted with Canada balsam (Junsei Chemical Co., Ltd., Tokyo, Japan; Cat# 23255S1210).

### 2.8. Western Blot Analyses

HK-2 cells and mice kidney tissue samples were analyzed as described previously. In short, protein extraction was performed using a lysis buffer comprising 1M PBS, 5 mM EDTA, and 0.5% Triton X-100. Samples underwent centrifugation at 13,000 rpm for 10 min, after which the clear supernatants were collected for subsequent Western blot analyses.

Extracted proteins were separated using SDS-PAGE and subsequently transferred onto PVDF membranes. Following transfer, membranes were blocked for 45 min at room temperature using a blocking solution containing 5% skim milk dissolved in Tris-buffered saline and supplemented with 0.1% Tween 20. Membranes were then incubated overnight at 4 °C with primary antibodies, including α-tubulin (Cell Signaling Technology, Danvers, MA, USA; Cat# 2144), α-SMA (Abcam, ab5694), PGC-1α (Abcam, ab54481), collagen Ⅳ (Abcam, ab6586), HIF-1(Abcam, ab179483), pERK (Cell Signaling Technology, Danvers, MA, USA; Cat# 9102), t-ERK (Santa Cruz Biotechnology, Dallas, TX, USA; Cat# sc-514302), and GAPDH (Santa Cruz Biotechnology, Dallas, TX, USA; Cat# sc-47724). Secondary antibodies (1:2000 dilution) were incubated for 1.5 h at RT. Finally, all the signals were visualized and analyzed using a bioanalytical imaging system.

### 2.9. Statistical Analyses

All data were analyzed using GraphPad Prism 9.0 and are indicated as the mean ± standard deviation of the mean values. Data were analyzed using a one-way analysis of variance with Tukey’s multiple comparisons post hoc test for comparing the mean values of multiple groups. *p* < 0.05 was considered statistically significant.

## 3. Results

### 3.1. Hypothermia Improves Renal Function

**Figure 2:**
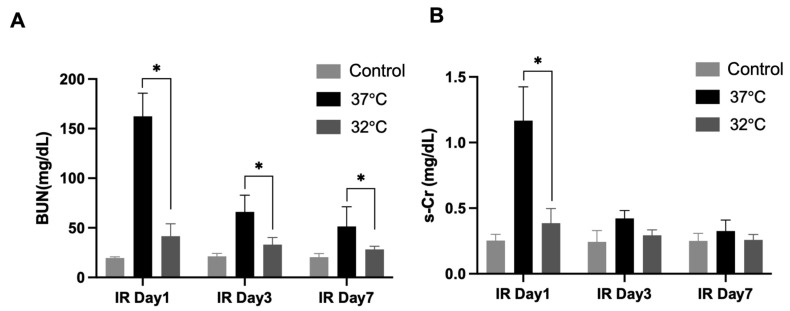
The effects of hypothermia on renal function with renal IR mice. Blood urea nitrogen (BUN) and serum creatinine (s-Cr) levels evaluated for renal function in the 37 °C group and 32 °C group, respectively, on days 1, 3, and 7 compared to control mice. *n* = 4–6 per group. (**A**) BUN levels were elevated in the 37 °C IRI group on days 1, 3, and 7 compared to controls, but significantly reduced in the 32 °C group at all time points relative to the 37 °C group (Figure 2A). (**B**) s-Cr levels peaked in the 37 °C group on day 1, while the 32 °C group showed lower levels than the 37 °C group at the same time point. The bars represent means ± SD. * *p* < 0.05.

For identifying the role of hypothermia in IRI on renal function, the mice’s s-creatinine (s-Cr) levels and blood urea nitrogen (BUN) levels were measured (Figure 2). The BUN levels were significantly higher in the 37 °C IRI group on day 1, day 3, and day 7 than in the control group, while they were significantly decreased in the 32 °C IRI group on day 1, day 3, and day 7 (Figure 2A) compared to the 37 °C IRI group on day 1, day 3, and day 7. The s-Cr levels increased most significantly in the 37 °C IRI group on day 1, compared to the control group. The s-Cr levels decreased in the 32 °C IRI group compared to the 37 °C IRI group on day 1 (Figure 2B).

### 3.2. Hypothermia Ameliorates the Progress of Renal Fibrosis After Ischemia–Reperfusion Injury of Mice Kidney

**Figure 3:**
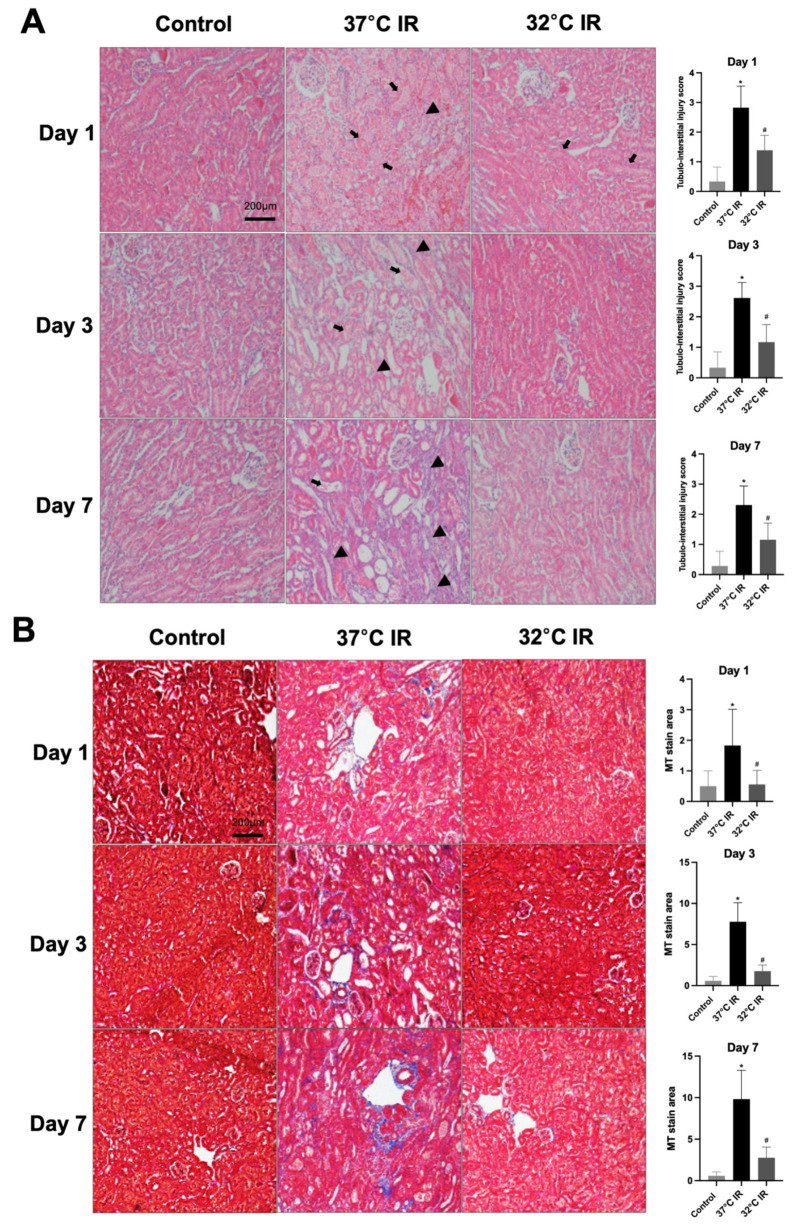
Hypothermic effect on renal histology. (**A**) Representative H and E stain of kidney section. Black arrows represent cell debris and tubular necrosis. Black arrowheads represent inflammatory cells. Original magnification, 200×. (**B**) Representative Masson’s trichrome stain of kidney section. Original magnification, 200×. * *p* < 0.05 vs. control group, ^#^ *p* < 0.05 vs. 37 °C IRI group. The bars represent means ± SD.

**Figure 4:**
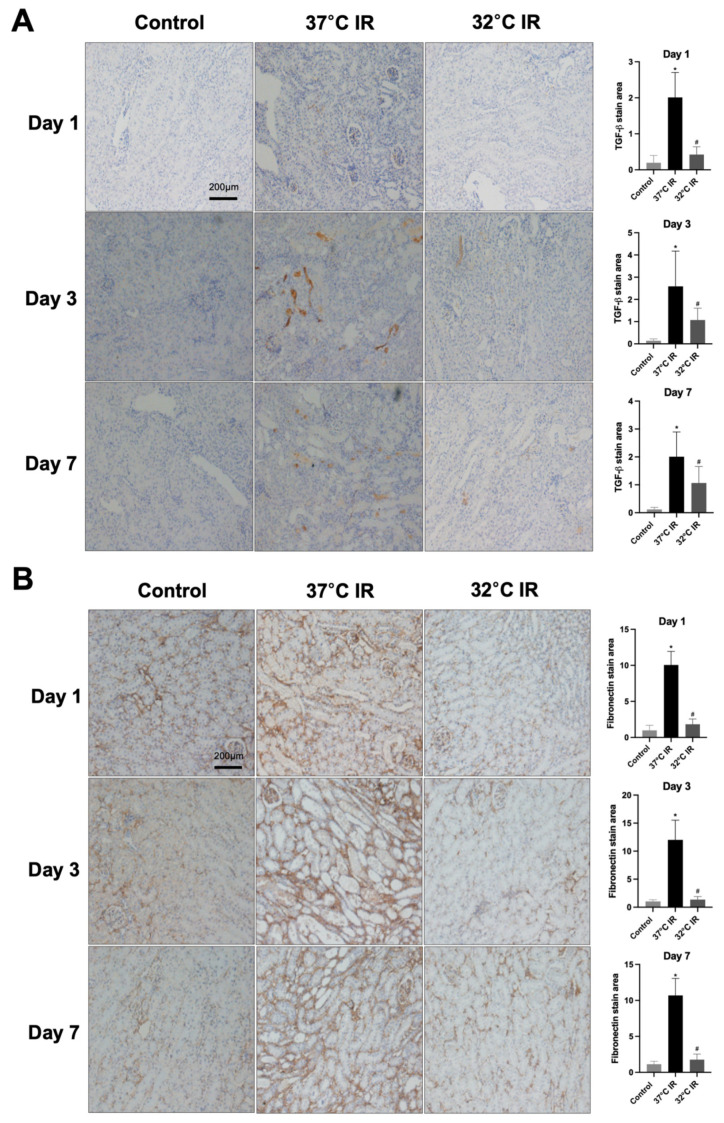

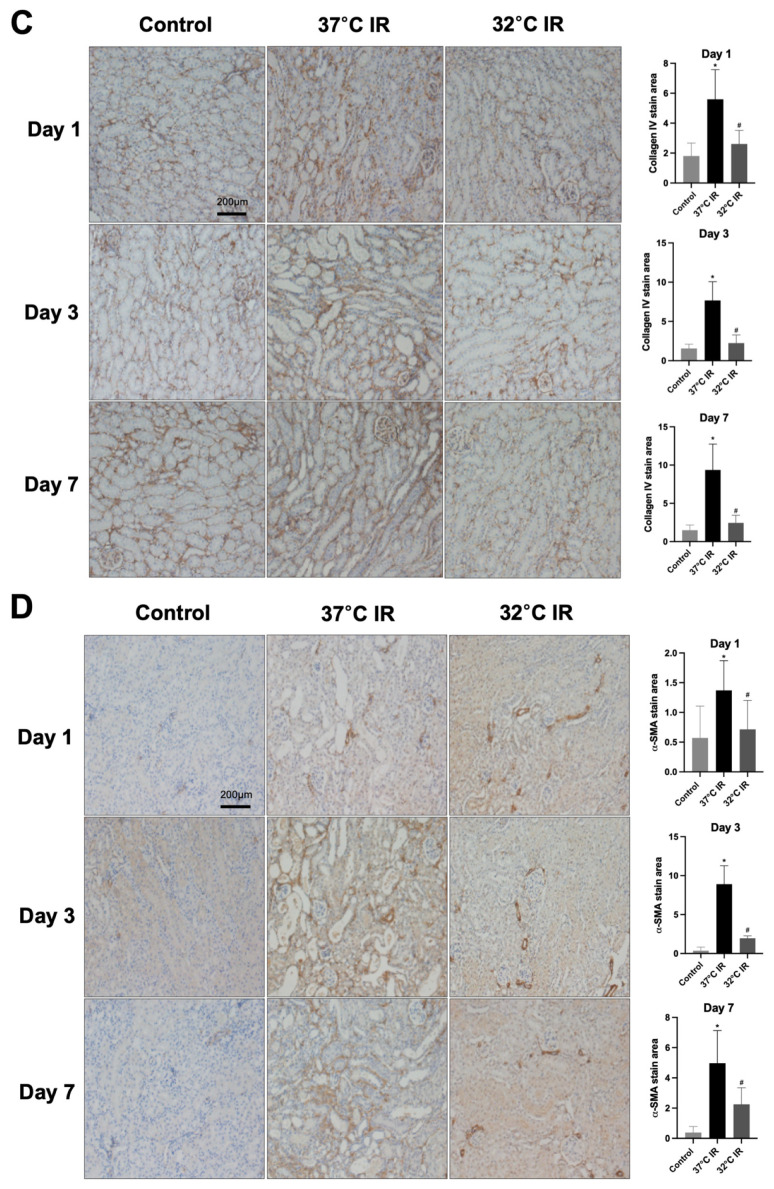
Immunostaining of renal fibrosis evaluation. (**A**) Representative TGF-β stain of kidney section and quantitative analysis. (**B**) Representative fibronectin stain of kidney section and quantitative analysis. (**C**) Representative collagen Ⅳ stain of kidney section and quantitative analysis. (**D**) Representative α-SMA stain of kidney section and quantitative analysis. Original magnification, 200×. * *p* < 0.05 vs. control group, ^#^ *p* < 0.05 vs. 37 °C IR group. The bars represent means ± SD.

**Figure 5:**
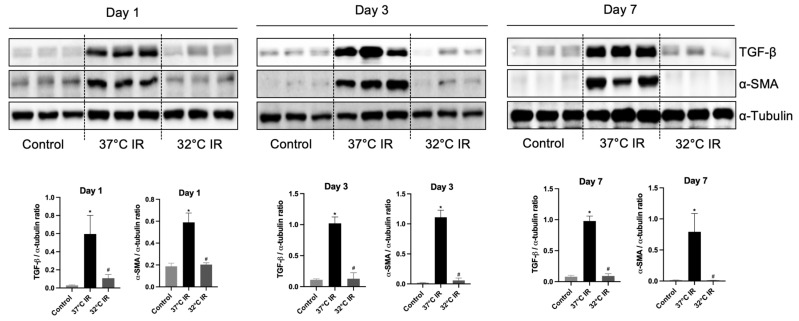
Representative images of Western blot and quantitative analysis of renal fibrosis markers. The protein levels of TGF-β1 and α-SMA in the kidney tissues of each experimental group were evaluated using Western blot. * *p* < 0.05 vs. control group, ^#^ *p* < 0.05 vs. 37 °C IR group. The bars represent means ± SD.

Histological analysis using H and E staining revealed significantly increased renal injury scores, including renal tubular cell atrophy, interstitial inflammation, and tubulointerstitial fibrosis, in the 37 °C IRI group compared to the control group. However, these injury scores were significantly lower in the hypothermic 32 °C IRI group (Figure 3A). Similarly, Masson’s trichrome staining indicated a significantly larger fibrotic area in the 37 °C IRI group compared to the control group, which was notably reduced in the hypothermic 32 °C IRI group (Figure 3B). Immunohistochemistry demonstrated significantly increased staining for TGF-β1, fibronectin, collagen IV, and α-SMA in the 37 °C IRI group compared to the control group’s kidneys (Figure 4A–D). These fibrotic markers were significantly decreased in all hypothermic 32 °C IRI groups at each measured time point (day 1, day 3, and day 7). Western blot analysis was performed to quantitatively evaluate fibrotic markers, including TGF-β1 and α-SMA in kidney tissues (Figure 5). The protein levels of TGF-β1 and α-SMA were significantly higher in the 37 °C IRI group on day 1, day 3, and day 7 than in the control group. However, these markers were significantly reduced in the 32 °C IRI group compared to the 37 °C IRI group on day 1, day 3, and day 7 (Figure 5). Collectively, these results suggest that hypothermia significantly attenuates renal fibrosis following IRI in mouse kidneys.

### 3.3. Hypothermia Increases the PGC-1α in Ischemia–Reperfusion Injury Mice Kidney

**Figure 6:**
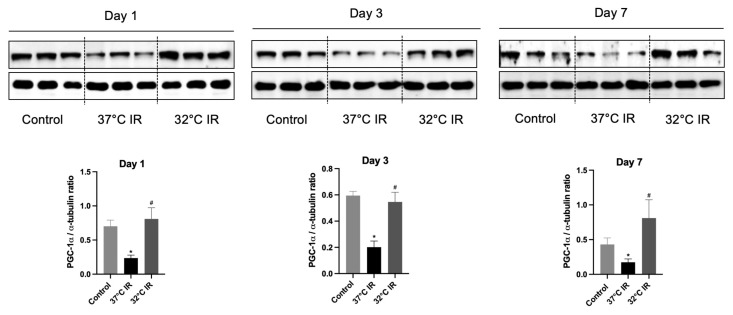
Representative images of Western blot and quantitative analysis of PGC-1α. The protein levels of TGF-β1 and α-SMA in the kidney tissues of each experimental group were evaluated using Western blot. * *p* < 0.05 vs. control group, ^#^ *p* < 0.05 vs. 37 °C IR group. The bars represent means ± SD.

In our previous study, we demonstrated that the hypothermia-induced activation of ERK and HIF plays an important role in attenuating acute kidney injury [17]. However, in the current model of renal fibrosis after renal IRI, we observed that ERK and HIF did not exhibit a significant protective role (Figure S1). We focused on PGC-1α, a transcriptional coactivator known to be robustly induced by hypothermia, which facilitates the expression of multiple protective proteins as part of the cellular defense mechanism under hypothermic conditions [20,21]. PGC-1α acts as a master regulator of mitochondrial function and is recognized for its protective role in kidney tissues. Previous reports have indicated that PGC-1α expression is disrupted in IRI animal models [16,20]. To evaluate the protective role of PGC-1α, we assessed its protein expression in a hypothermic (32 °C) IRI mouse model. Compared to the control group, PGC-1α expression was significantly reduced in normothermic (37 °C) IRI groups on days 1, 3, and 7. Conversely, PGC-1α levels were significantly increased in all hypothermic IRI groups at the same time points, compared to the normothermic IRI groups (Figure 6). Thus, our findings suggest that hypothermia confers renal protection by preserving PGC-1α expression following renal IRI.

### 3.4. Knockdown of PGC-1α Expression Induces Renal Fibrosis in TGF-β1-Treated HK-2 Cells

**Figure 7:**
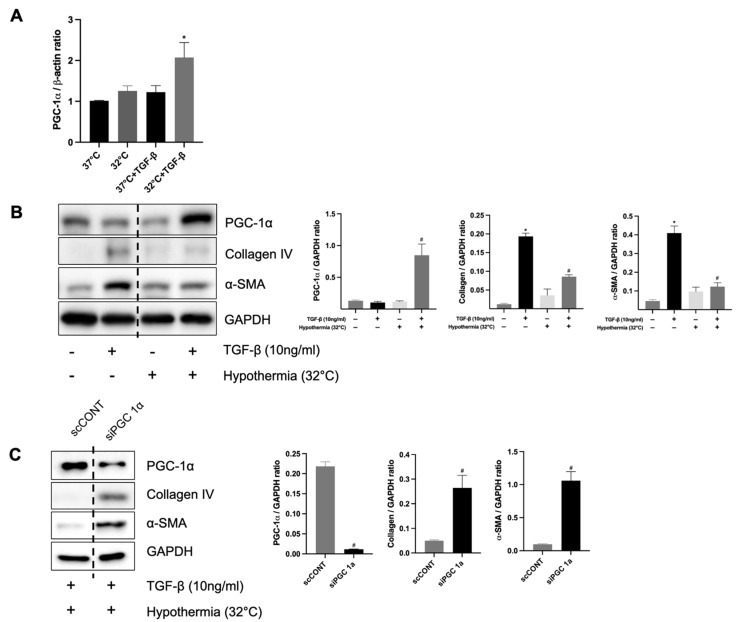
The protective role of hypothermia on renal fibrosis and PGC-1α in vitro. (**A**) The mRNA expression of PGC-1α; * *p* < 0.05 vs. 37 °C + TGF-β group. (**B**) Protein expression levels of PGC-1α, collagen IV, and α-SMA in TGF-β-treated HK-2 cells under hypothermic (32 °C) and normothermic conditions (37 °C); * *p* < 0.05 vs. 37 °C without TGF-β group, ^#^ *p* < 0.05 vs. 37 °C with TGF-β group. (**C**) Protein expression levels of α-SMA and collagen IV under TGF-β-treated hypothermic conditions (32 °C) in HK-2 cells with or without knockdown of PGC-1α; ^#^ *p* < 0.05 vs. scCONT group. The bars represent means ± SD.

To clarify the relationship between hypothermia-induced PGC-1α expression and renal fibrosis in vitro, HK-2 cells were incubated at 32 °C and 37 °C using an incubator for 24 h. The mRNA expression levels of PGC-1α were significantly increased in TGF-β-treated HK-2 cells at 32 °C compared to TGF-β-treated cells at 37 °C. (Figure 7A). The protein levels of collagen IV and α-SMA were decreased in TGF-β-treated HK-2 cells at 32 °C compared to those at 37 °C. The protein levels of PGC-1α were increased in TGF-β-treated HK-2 cells at 32 °C compared to those at 37 °C (Figure 7B). These results showed that hypothermia increases PGC-1α in TGF-β-treated HK-2 cells and decreases fibrosis. Furthermore, to determine the functional role of PGC-1α in renal fibrosis, PGC-1α expression was knocked down using siRNA in HK-2 cells treated with TGF-β1. Western blot analysis demonstrated significantly increased protein expressions of collagen IV and α-SMA in HK-2 cells transfected with PGC-1α siRNA, compared to those transfected with non-targeting control siRNA (Figure 7C). These findings confirm that the knockdown of PGC-1α exacerbates fibrosis in TGF-β1-treated HK-2 cells, underscoring the protective role of PGC-1α against renal fibrosis.

## 4. Discussion

This study demonstrates that hypothermia regulates fibrosis through PGC-1α. The experimental data clearly demonstrate that hypothermic treatment significantly attenuated renal injury and preserved renal histoarchitecture, which collectively contributed to the suppression of fibrosis. Notably, the renoprotective effect of hypothermia was abrogated when PGC-1α expression was silenced using siRNA, thereby underscoring the pivotal role of PGC-1α in this protective pathway. These findings suggest that targeting PGC-1α activation could represent a promising strategy to preserve renal function in the context of injury.

In the current study, renal fibrosis was experimentally induced in mice via ischemia–reperfusion injury (IRI), a well-established model that mimics clinical renal pathologies. Consistent with prior reports [22], markers of fibrosis were markedly elevated in the IRI-induced normothermia group. Conversely, hypothermic treatment markedly reduced renal damage and fibrosis severity.

In comparing our results with previous research, our study contributes novel insights into the renoprotective mechanisms of hypothermia, particularly in the context of renal fibrosis. Previously, our research identified that ERK is an important factor that plays a role in reducing renal injury [17]. However, the ERK activation pattern observed in the current fibrosis model differs from that seen in our previous acute IRI model. Specifically, while ERK was upregulated under hypothermic conditions in the acute model (i.e., day 1 after IRI), in the fibrosis model (i.e., days 3 and 7 after IRI), ERK expression was higher in the IRI normothermia group than in the hypothermia group. This observation aligns with previous reports indicating that ERK activation contributes to the progression of fibrosis in chronic kidney injury models [23,24]. This observation supports recent studies reporting that sustained ERK activation contributes to fibrotic progression rather than repair, highlighting the context-dependent duality of ERK signaling.

PGC-1α is a member of a family of transcription cold-inducible coactivators that plays a central role in regulating cellular energy metabolism processes, such as mitochondrial and peroxisomal biogenesis [25,26]. It is strongly induced by cold exposure [11,27,28]. PGC-1α expression is localized to the cortex and the outer stripe of the medulla, overlapping with regions of high mitochondrial activity in the kidney. There are relatively more mitochondria in the kidneys than in other organs [29]. Emerging evidence suggests that the loss of PGC-1α following acute kidney injury (AKI) contributes to the development of fibrosis by disrupting mitochondrial homeostasis, and its restoration is believed to be crucial for kidney repair and recovery [29].

Our data demonstrate that hypothermia increases the PGC-1α and ameliorates tubule interstitial fibrosis in IRI mice. In contrast, normothermic conditions were associated with reduced PGC-1α levels and aggravated fibrotic changes. These findings are in harmony with earlier studies, indicating that diminished PGC-1α expression is a key driver of fibrotic development. To corroborate these in vivo findings, we employed an in vitro model using human kidney (HK-2) cells treated with transforming growth factor-beta 1 (TGF-β1), a well-known inducer of fibrosis. The knockdown of PGC-1α in this setting led to the increased expression of fibrotic markers, thereby validating its protective role. Therefore, this is consistent with other studies that have stated that the loss of PGC-1α contributes to fibrosis development [29].

Moreover, we knocked down PGC-1α in vitro to clarify that PGC-1α plays a protective role in renal fibrosis. The siRNA of PGC-1α increases the level of renal fibrosis markers in TGF-β1-treated HK-2 cells, supporting the theory that PGC-1α reduces renal fibrosis. Choi et al. showed that PGC-1α suppresses TGF-β/Smad signaling by targeting TGFβR1 [30].

Previous research by Choi et al. [30] has shown that PGC-1α can inhibit TGF-β/Smad signaling by targeting TGF-β receptor type I (TGFβR1). In line with their findings, our study reveals that hypothermia-induced PGC-1α expression interferes with pro-fibrotic pathways at multiple levels. First, hypothermia enhances mitochondrial function and reduces oxidative stress by preserving mitochondrial homeostasis through PGC-1α upregulation. Second, PGC-1α downregulates the TGF-β/Smad axis, a principal fibrogenic pathway, likely via the suppression of TGFβR1 expression. Notably, Choi et al. [30] also suggested a microRNA-mediated mechanism, in which PGC-1α modulates let-7b/c miRNA levels to repress TGFβR1. Third, our in vitro findings clearly demonstrate that, under hypothermic conditions, PGC-1α diminishes the expression of fibrosis-related markers, such as alpha-smooth muscle actin (α-SMA) and collagen IV, even in the presence of TGF-β1.

A strength of this study is that it is the first to demonstrate that hypothermia alleviates renal fibrosis following ischemia–reperfusion injury through the upregulation of PGC-1α, thereby establishing a novel molecular connection between cold-induced protection and mitochondrial regulation in the kidney. Additionally, the use of both in vivo and in vitro models enhances the translational relevance of the findings and provides a comprehensive mechanistic insight into the antifibrotic effects of hypothermia.

Despite the robustness of these findings, this study has some limitations. First, longitudinal comparisons among the 1-, 3-, and 7-day time points after IRI were not performed, which limits our ability to thoroughly characterize the progression of fibrosis and the degree of its inhibition over time. Second, our data may not reflect the peak stage of fibrosis because observations beyond 7 days post-IRI were not conducted. Therefore, concerns remain regarding whether hypothermia differentially improves various stages of fibrotic progression. Third, our study did not successfully establish a hypoxia–reoxygenation-induced cellular fibrosis model comparable to the animal model; therefore, a TGF-β treatment model was employed. To clarify these differences more precisely, future studies using PGC-1α knockout (KO) mice will be necessary. Fourth, our findings are limited to in vivo models, and due to the absence of patient-derived samples or clinical correlation analyses, further clinical studies are needed to directly validate the proposed molecular mechanisms.

## 5. Conclusions

This study provides evidence that hypothermia reduces renal fibrosis, highlighting PGC-1α as a key molecular mediator in this process. The therapeutic effects of hypothermia appear to arise from its ability to preserve mitochondrial homeostasis, inhibit oxidative stress, and downregulate fibrotic signaling pathways, particularly those driven by TGF-β/Smad. Importantly, our data show that silencing PGC-1α abolishes these beneficial effects, suggesting that PGC-1α is not merely associated with but is essential for hypothermia-mediated protection. Although the underlying mechanisms of hypothermia remain incompletely understood, our findings establish a strong rationale for exploring PGC-1α as a potential therapeutic target in renal fibrosis. Future investigations should focus on elucidating the downstream effectors of PGC-1α and evaluating the efficacy of pharmacological agents that can mimic or enhance its activity in the context of renal injury and fibrosis.

## Data Availability

The original contributions presented in this study are included in the article/Supplementary Material. Further inquiries can be directed to the corresponding author.

## Supplementary Materials

The following supporting information can be downloaded at: https://www.mdpi.com/article/10.3390/biomedicines13061337/s1. Figure S1. Representative images of western blot and quantitative analysis of ERK and HIF1. The protein levels of pERK and HIF1α were analyzed using western blot. On Day 1, ERK and HIF1 expression levels in the 32 °C IR group were similar to or higher than those in the 37 °C IR group. However, on Days 3 and 7, when renal fibrotic injury occurred, ERK and HIF1 expression significantly decreased in the 32°C IR group compared to the 37 °C IR group. * *p* < 0.05 vs. Control group, ^#^ *p* < 0.05 vs. 37 °C IR group. The bars represent means ± SD.
