# Supplementary material for: Hypothermia Mitigates Renal Fibrosis Through the Upregulation of PGC-1α After Ischemia–Reperfusion Injury
Source: Biomedicines. 2025 May 29;13(6):1337. doi: 10.3390/biomedicines13061337 (PMC12189753; doi:10.3390/biomedicines13061337)
Supplement: Supplementary file 1 [file biomedicines-13-01337-s001.zip › biomedicines-3586366-supplementary.pdf]

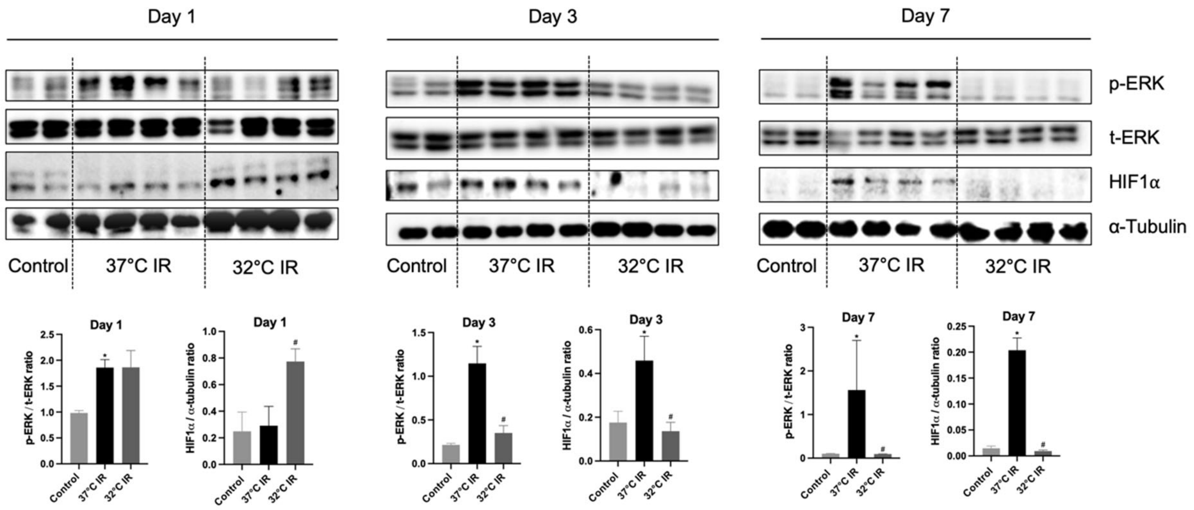

Figure S1. Representative images of western blot and quantitative analysis of ERK and HIF1. The protein levels of pERK and HIF1**α** were analyzed using western blot. On Day 1, ERK and HIF1 expression levels in the 32°C IR group were similar to or higher than those in the 37°C IR group. However, on Days 3 and 7, when renal fibrotic injury occurred, ERK and HIF1 expression significantly decreased in the 32°C IR group compared to the 37°C IR group. *P<0.05 vs. Control group, $^{\#}P$<0.05 vs. 37°C IR group. The bars represent means ± SD.
